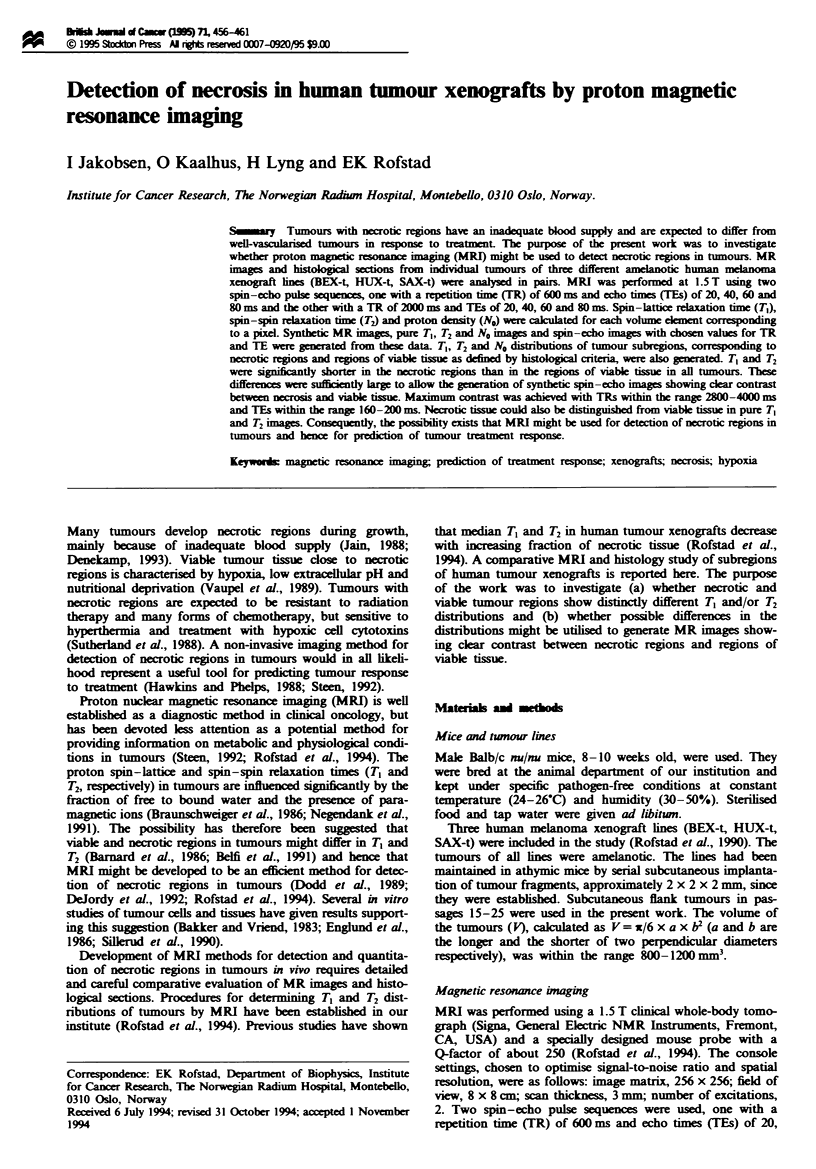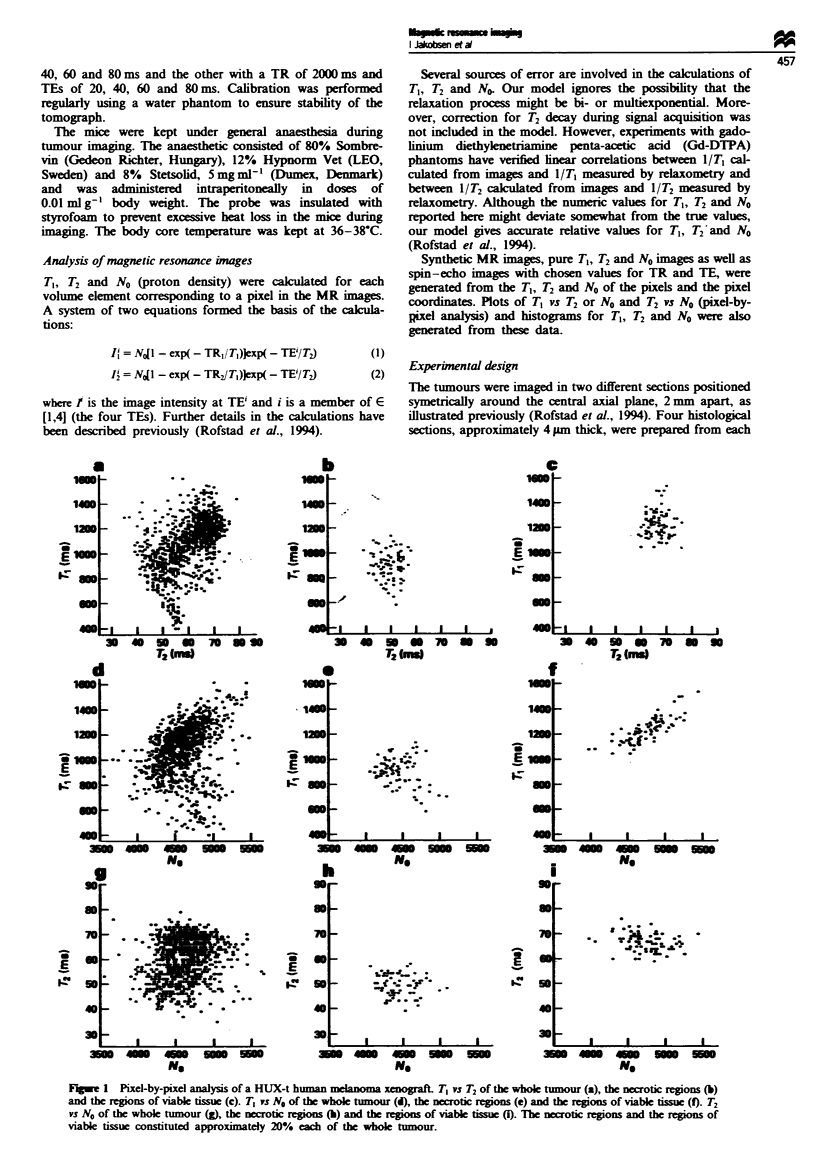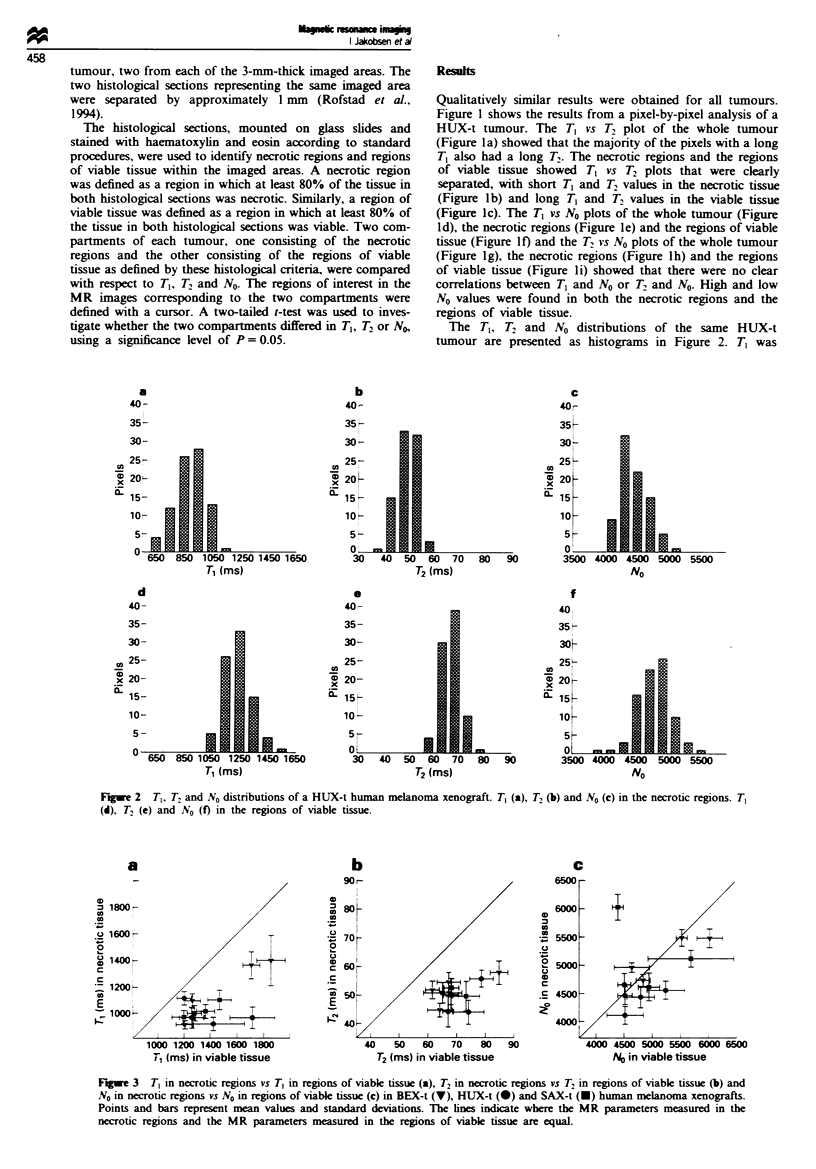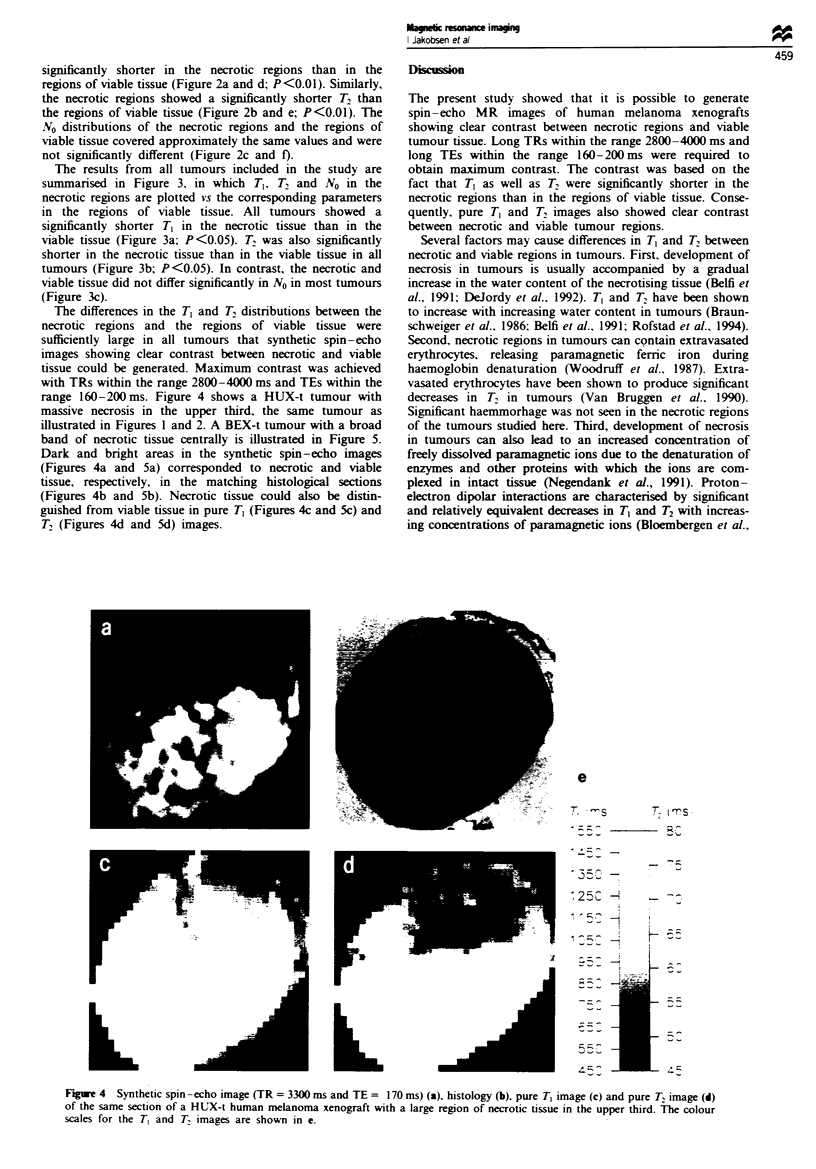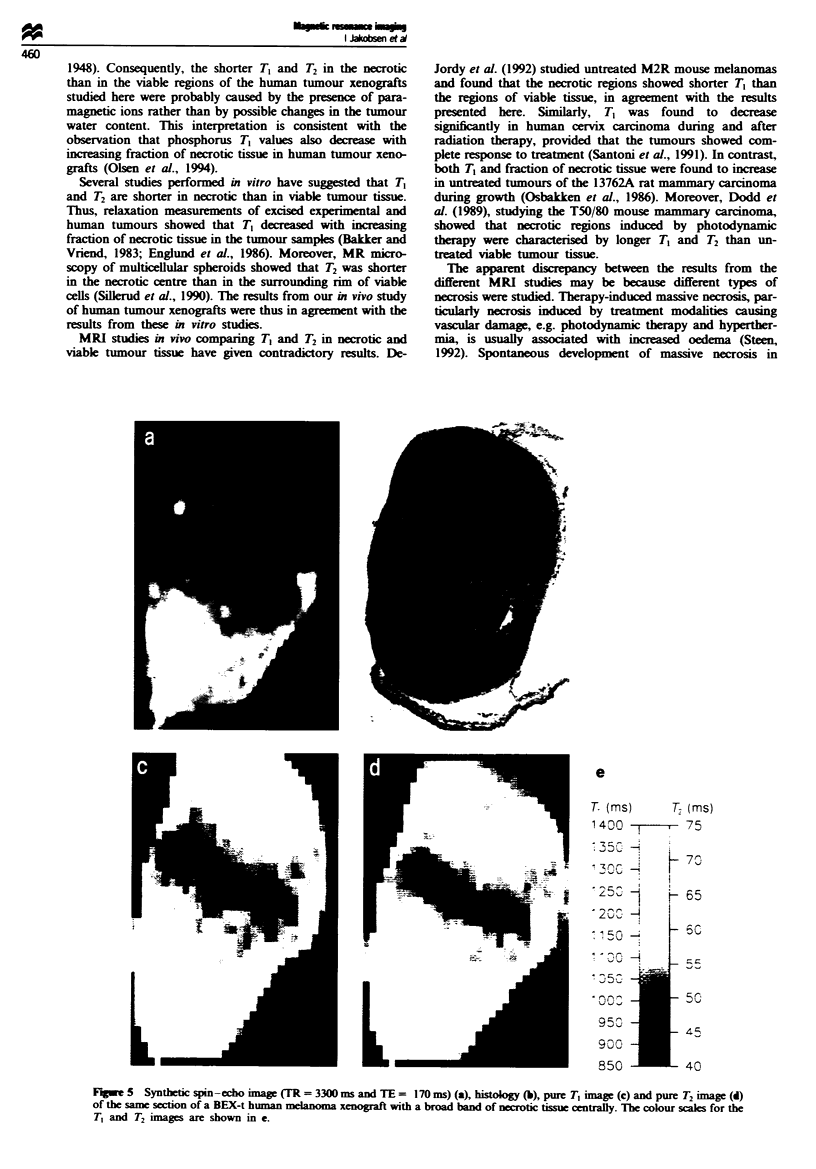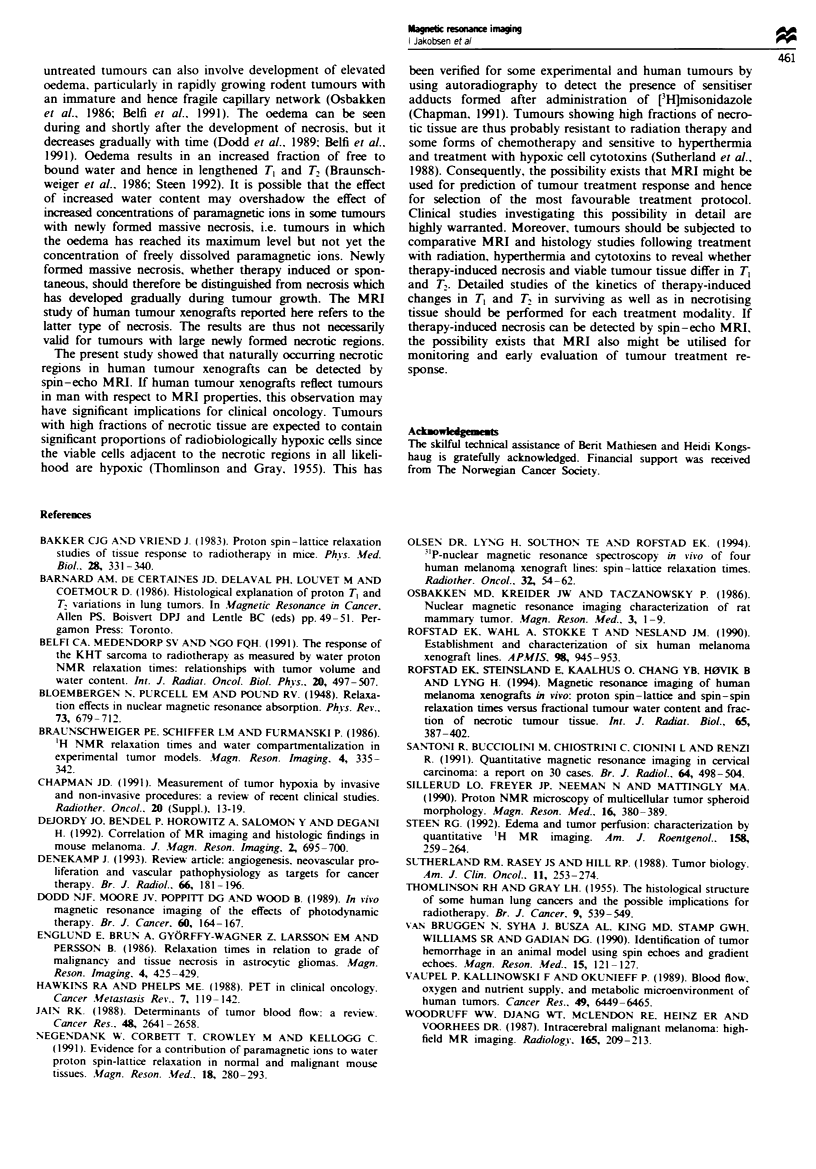# Detection of necrosis in human tumour xenografts by proton magnetic resonance imaging.

**DOI:** 10.1038/bjc.1995.93

**Published:** 1995-03

**Authors:** I. Jakobsen, O. Kaalhus, H. Lyng, E. K. Rofstad

**Affiliations:** Institute for Cancer Research, Norwegian Radium Hospital, Montebello, Oslo.

## Abstract

**Images:**


### Detection of necrosis in human tumour xenografts by proton magnetic resonance imaging

I Jakobsen, O Kaalhus, H Lyng and EK Rofstad

*Institute for Cancer Research, The Norwegian Radium Hospital, Montebello, 0310 Oslo, Norway.*

**Summary** Tumours with necrotic regions have an inadequate blood supply and are expected to differ from well-vascularised tumours in response to treatment. The purpose of the present work was to investigate whether proton magnetic resonance imaging (MRI) might be used to detect necrotic regions in tumours. MR images and histological sections from individual tumours of three different amelanotic human melanoma xenograft lines (BEX-t, HUX-t, SAX-t) were analysed in pairs. MRI was performed at 1.5 T using two spin–echo pulse sequences, one with a repetition time (TR) of 600 ms and echo times (TEs) of 20, 40, 60 and 80 ms and the other with a TR of 2000 ms and TEs of 20, 40, 60 and 80 ms. Spin–lattice relaxation time ($T_1$), spin–spin relaxation time ($T_2$) and proton density ($N_0$) were calculated for each volume element corresponding to a pixel. Synthetic MR images, pure $T_1$, $T_2$ and $N_0$ images and spin–echo images with chosen values for TR and TE were generated from these data. $T_1$, $T_2$ and $N_0$ distributions of tumour subregions, corresponding to necrotic regions and regions of viable tissue as defined by histological criteria, were also generated. $T_1$ and $T_2$ were significantly shorter in the necrotic regions than in the regions of viable tissue in all tumours. These differences were sufficiently large to allow the generation of synthetic spin–echo images showing clear contrast between necrosis and viable tissue. Maximum contrast was achieved with TRs within the range 2800–4000 ms and TEs within the range 160–200 ms. Necrotic tissue could also be distinguished from viable tissue in pure $T_1$ and $T_2$ images. Consequently, the possibility exists that MRI might be used for detection of necrotic regions in tumours and hence for prediction of tumour treatment response.

**Keywords:** magnetic resonance imaging; prediction of treatment response; xenografts; necrosis; hypoxia

Many tumours develop necrotic regions during growth, mainly because of inadequate blood supply (Jain, 1988; Denekamp, 1993). Viable tumour tissue close to necrotic regions is characterised by hypoxia, low extracellular pH and nutritional deprivation (Vaupel *et al.*, 1989). Tumours with necrotic regions are expected to be resistant to radiation therapy and many forms of chemotherapy, but sensitive to hyperthermia and treatment with hypoxic cell cytotoxins (Sutherland *et al.*, 1988). A non-invasive imaging method for detection of necrotic regions in tumours would in all likelihood represent a useful tool for predicting tumour response to treatment (Hawkins and Phelps, 1988; Steen, 1992).

Proton nuclear magnetic resonance imaging (MRI) is well established as a diagnostic method in clinical oncology, but has been devoted less attention as a potential method for providing information on metabolic and physiological conditions in tumours (Steen, 1992; Rofstad *et al.*, 1994). The proton spin–lattice and spin–spin relaxation times ($T_1$ and $T_2$, respectively) in tumours are influenced significantly by the fraction of free to bound water and the presence of paramagnetic ions (Braunschweiger *et al.*, 1986; Negendank *et al.*, 1991). The possibility has therefore been suggested that viable and necrotic regions in tumours might differ in $T_1$ and $T_2$ (Barnard *et al.*, 1986; Belfi *et al.*, 1991) and hence that MRI might be developed to be an efficient method for detection of necrotic regions in tumours (Dodd *et al.*, 1989; DeJordy *et al.*, 1992; Rofstad *et al.*, 1994). Several *in vitro* studies of tumour cells and tissues have given results supporting this suggestion (Bakker and Vriend, 1983; Englund *et al.*, 1986; Sillerud *et al.*, 1990).

Development of MRI methods for detection and quantitation of necrotic regions in tumours *in vivo* requires detailed and careful comparative evaluation of MR images and histological sections. Procedures for determining $T_1$ and $T_2$ distributions of tumours by MRI have been established in our institute (Rofstad *et al.*, 1994). Previous studies have shown that median $T_1$ and $T_2$ in human tumour xenografts decrease with increasing fraction of necrotic tissue (Rofstad *et al.*, 1994). A comparative MRI and histology study of subregions of human tumour xenografts is reported here. The purpose of the work was to investigate (a) whether necrotic and viable tumour regions show distinctly different $T_1$ and/or $T_2$ distributions and (b) whether possible differences in the distributions might be utilised to generate MR images showing clear contrast between necrotic regions and regions of viable tissue.

Correspondence: EK Rofstad, Department of Biophysics, Institute for Cancer Research, The Norwegian Radium Hospital, Montebello, 0310 Oslo, Norway
Received 6 July 1994; revised 31 October 1994; accepted 1 November 1994

### Materials and methods

### *Mice and tumour lines*

Male Balb/c *nu/nu* mice, 8–10 weeks old, were used. They were bred at the animal department of our institution and kept under specific pathogen-free conditions at constant temperature (24–26°C) and humidity (30–50%). Sterilised food and tap water were given *ad libitum*.

Three human melanoma xenograft lines (BEX-t, HUX-t, SAX-t) were included in the study (Rofstad *et al.*, 1990). The tumours of all lines were amelanotic. The lines had been maintained in athymic mice by serial subcutaneous implantation of tumour fragments, approximately 2 × 2 × 2 mm, since they were established. Subcutaneous flank tumours in passages 15–25 were used in the present work. The volume of the tumours ($V$), calculated as $V = \pi/6 \times a \times b^2$ ($a$ and $b$ are the longer and the shorter of two perpendicular diameters respectively), was within the range 800–1200 mm$^3$.

### *Magnetic resonance imaging*

MRI was performed using a 1.5 T clinical whole-body tomograph (Signa, General Electric NMR Instruments, Fremont, CA, USA) and a specially designed mouse probe with a Q-factor of about 250 (Rofstad *et al.*, 1994). The console settings, chosen to optimise signal-to-noise ratio and spatial resolution, were as follows: image matrix, 256 × 256; field of view, 8 × 8 cm; scan thickness, 3 mm; number of excitations, 2. Two spin–echo pulse sequences were used, one with a repetition time (TR) of 600 ms and echo times (TEs) of 20,

40, 60 and 80 ms and the other with a TR of 2000 ms and TEs of 20, 40, 60 and 80 ms. Calibration was performed regularly using a water phantom to ensure stability of the tomograph.

The mice were kept under general anaesthesia during tumour imaging. The anaesthetic consisted of 80% Sombrevin (Gedeon Richter, Hungary), 12% Hypnorm Vet (LEO, Sweden) and 8% Stetsolid, 5 mg $ml^{-1}$ (Dumex, Denmark) and was administered intraperitoneally in doses of 0.01 ml $g^{-1}$ body weight. The probe was insulated with styrofoam to prevent excessive heat loss in the mice during imaging. The body core temperature was kept at 36–38°C.

*Analysis of magnetic resonance images*

$T_1$, $T_2$ and $N_0$ (proton density) were calculated for each volume element corresponding to a pixel in the MR images. A system of two equations formed the basis of the calculations:

$$I_1^i = N_0[1 - \exp(-TR_1/T_1)]\exp(-TE^i/T_2) \quad (1)$$

$$I_2^i = N_0[1 - \exp(-TR_2/T_1)]\exp(-TE^i/T_2) \quad (2)$$

where $I^i$ is the image intensity at $TE^i$ and $i$ is a member of ∈ [1,4] (the four TEs). Further details in the calculations have been described previously (Rofstad *et al.*, 1994).

Several sources of error are involved in the calculations of $T_1$, $T_2$ and $N_0$. Our model ignores the possibility that the relaxation process might be bi- or multiexponential. Moreover, correction for $T_2$ decay during signal acquisition was not included in the model. However, experiments with gadolinium diethylenetriamine penta-acetic acid (Gd-DTPA) phantoms have verified linear correlations between $1/T_1$ calculated from images and $1/T_1$ measured by relaxometry and between $1/T_2$ calculated from images and $1/T_2$ measured by relaxometry. Although the numeric values for $T_1$, $T_2$ and $N_0$ reported here might deviate somewhat from the true values, our model gives accurate relative values for $T_1$, $T_2$ and $N_0$ (Rofstad *et al.*, 1994).

Synthetic MR images, pure $T_1$, $T_2$ and $N_0$ images as well as spin–echo images with chosen values for TR and TE, were generated from the $T_1$, $T_2$ and $N_0$ of the pixels and the pixel coordinates. Plots of $T_1$ *vs* $T_2$ or $N_0$ and $T_2$ *vs* $N_0$ (pixel-by-pixel analysis) and histograms for $T_1$, $T_2$ and $N_0$ were also generated from these data.

*Experimental design*

The tumours were imaged in two different sections positioned symetrically around the central axial plane, 2 mm apart, as illustrated previously (Rofstad *et al.*, 1994). Four histological sections, approximately 4 μm thick, were prepared from each

**Figure 1** Pixel-by-pixel analysis of a HUX-t human melanoma xenograft. $T_1$ *vs* $T_2$ of the whole tumour (**a**), the necrotic regions (**b**) and the regions of viable tissue (**c**). $T_1$ *vs* $N_0$ of the whole tumour (**d**), the necrotic regions (**e**) and the regions of viable tissue (**f**). $T_2$ *vs* $N_0$ of the whole tumour (**g**), the necrotic regions (**h**) and the regions of viable tissue (**i**). The necrotic regions and the regions of viable tissue constituted approximately 20% each of the whole tumour.

tumour, two from each of the 3-mm-thick imaged areas. The two histological sections representing the same imaged area were separated by approximately 1 mm (Rofstad *et al.*, 1994).

The histological sections, mounted on glass slides and stained with haematoxylin and eosin according to standard procedures, were used to identify necrotic regions and regions of viable tissue within the imaged areas. A necrotic region was defined as a region in which at least 80% of the tissue in both histological sections was necrotic. Similarly, a region of viable tissue was defined as a region in which at least 80% of the tissue in both histological sections was viable. Two compartments of each tumour, one consisting of the necrotic regions and the other consisting of the regions of viable tissue as defined by these histological criteria, were compared with respect to $T_1$, $T_2$ and $N_0$. The regions of interest in the MR images corresponding to the two compartments were defined with a cursor. A two-tailed $t$-test was used to investigate whether the two compartments differed in $T_1$, $T_2$ or $N_0$, using a significance level of $P = 0.05$.

### Results

Qualitatively similar results were obtained for all tumours. Figure 1 shows the results from a pixel-by-pixel analysis of a HUX-t tumour. The $T_1$ *vs* $T_2$ plot of the whole tumour (Figure 1a) showed that the majority of the pixels with a long $T_1$ also had a long $T_2$. The necrotic regions and the regions of viable tissue showed $T_1$ *vs* $T_2$ plots that were clearly separated, with short $T_1$ and $T_2$ values in the necrotic tissue (Figure 1b) and long $T_1$ and $T_2$ values in the viable tissue (Figure 1c). The $T_1$ *vs* $N_0$ plots of the whole tumour (Figure 1d), the necrotic regions (Figure 1e) and the regions of viable tissue (Figure 1f) and the $T_2$ *vs* $N_0$ plots of the whole tumour (Figure 1g), the necrotic regions (Figure 1h) and the regions of viable tissue (Figure 1i) showed that there were no clear correlations between $T_1$ and $N_0$ or $T_2$ and $N_0$. High and low $N_0$ values were found in both the necrotic regions and the regions of viable tissue.

The $T_1$, $T_2$ and $N_0$ distributions of the same HUX-t tumour are presented as histograms in Figure 2. $T_1$ was

**Figure 2** $T_1$, $T_2$ and $N_0$ distributions of a HUX-t human melanoma xenograft. $T_1$ (**a**), $T_2$ (**b**) and $N_0$ (**c**) in the necrotic regions. $T_1$ (**d**), $T_2$ (**e**) and $N_0$ (**f**) in the regions of viable tissue.

**Figure 3** $T_1$ in necrotic regions *vs* $T_1$ in regions of viable tissue (**a**), $T_2$ in necrotic regions *vs* $T_2$ in regions of viable tissue (**b**) and $N_0$ in necrotic regions *vs* $N_0$ in regions of viable tissue (**c**) in BEX-t (▼), HUX-t (●) and SAX-t (■) human melanoma xenografts. Points and bars represent mean values and standard deviations. The lines indicate where the MR parameters measured in the necrotic regions and the MR parameters measured in the regions of viable tissue are equal.

significantly shorter in the necrotic regions than in the regions of viable tissue (Figure 2a and d; $P<0.01$). Similarly, the necrotic regions showed a significantly shorter $T_2$ than the regions of viable tissue (Figure 2b and e; $P<0.01$). The $N_0$ distributions of the necrotic regions and the regions of viable tissue covered approximately the same values and were not significantly different (Figure 2c and f).

The results from all tumours included in the study are summarised in Figure 3, in which $T_1$, $T_2$ and $N_0$ in the necrotic regions are plotted *vs* the corresponding parameters in the regions of viable tissue. All tumours showed a significantly shorter $T_1$ in the necrotic tissue than in the viable tissue (Figure 3a; $P<0.05$). $T_2$ was also significantly shorter in the necrotic tissue than in the viable tissue in all tumours (Figure 3b; $P<0.05$). In contrast, the necrotic and viable tissue did not differ significantly in $N_0$ in most tumours (Figure 3c).

The differences in the $T_1$ and $T_2$ distributions between the necrotic regions and the regions of viable tissue were sufficiently large in all tumours that synthetic spin-echo images showing clear contrast between necrotic and viable tissue could be generated. Maximum contrast was achieved with TRs within the range 2800-4000 ms and TEs within the range 160-200 ms. Figure 4 shows a HUX-t tumour with massive necrosis in the upper third, the same tumour as illustrated in Figures 1 and 2. A BEX-t tumour with a broad band of necrotic tissue centrally is illustrated in Figure 5. Dark and bright areas in the synthetic spin-echo images (Figures 4a and 5a) corresponded to necrotic and viable tissue, respectively, in the matching histological sections (Figures 4b and 5b). Necrotic tissue could also be distinguished from viable tissue in pure $T_1$ (Figures 4c and 5c) and $T_2$ (Figures 4d and 5d) images.

### Discussion

The present study showed that it is possible to generate spin-echo MR images of human melanoma xenografts showing clear contrast between necrotic regions and viable tumour tissue. Long TRs within the range 2800-4000 ms and long TEs within the range 160-200 ms were required to obtain maximum contrast. The contrast was based on the fact that $T_1$ as well as $T_2$ were significantly shorter in the necrotic regions than in the regions of viable tissue. Consequently, pure $T_1$ and $T_2$ images also showed clear contrast between necrotic and viable tumour regions.

Several factors may cause differences in $T_1$ and $T_2$ between necrotic and viable regions in tumours. First, development of necrosis in tumours is usually accompanied by a gradual increase in the water content of the necrotising tissue (Belfi *et al.*, 1991; DeJordy *et al.*, 1992). $T_1$ and $T_2$ have been shown to increase with increasing water content in tumours (Braunschweiger *et al.*, 1986; Belfi *et al.*, 1991; Rofstad *et al.*, 1994). Second, necrotic regions in tumours can contain extravasated erythrocytes, releasing paramagnetic ferric iron during haemoglobin denaturation (Woodruff *et al.*, 1987). Extravasated erythrocytes have been shown to produce significant decreases in $T_2$ in tumours (Van Bruggen *et al.*, 1990). Significant haemmorhage was not seen in the necrotic regions of the tumours studied here. Third, development of necrosis in tumours can also lead to an increased concentration of freely dissolved paramagnetic ions due to the denaturation of enzymes and other proteins with which the ions are complexed in intact tissue (Negendank *et al.*, 1991). Proton-electron dipolar interactions are characterised by significant and relatively equivalent decreases in $T_1$ and $T_2$ with increasing concentrations of paramagnetic ions (Bloembergen *et al.*,

**Figure 4** Synthetic spin-echo image (TR = 3300 ms and TE = 170 ms) (**a**), histology (**b**), pure $T_1$ image (**c**) and pure $T_2$ image (**d**) of the same section of a HUX-t human melanoma xenograft with a large region of necrotic tissue in the upper third. The colour scales for the $T_1$ and $T_2$ images are shown in **e**.

1948). Consequently, the shorter $T_1$ and $T_2$ in the necrotic than in the viable regions of the human tumour xenografts studied here were probably caused by the presence of paramagnetic ions rather than by possible changes in the tumour water content. This interpretation is consistent with the observation that phosphorus $T_1$ values also decrease with increasing fraction of necrotic tissue in human tumour xenografts (Olsen *et al.*, 1994).

Several studies performed *in vitro* have suggested that $T_1$ and $T_2$ are shorter in necrotic than in viable tumour tissue. Thus, relaxation measurements of excised experimental and human tumours showed that $T_1$ decreased with increasing fraction of necrotic tissue in the tumour samples (Bakker and Vriend, 1983; Englund *et al.*, 1986). Moreover, MR microscopy of multicellular spheroids showed that $T_2$ was shorter in the necrotic centre than in the surrounding rim of viable cells (Sillerud *et al.*, 1990). The results from our *in vivo* study of human tumour xenografts were thus in agreement with the results from these *in vitro* studies.

MRI studies *in vivo* comparing $T_1$ and $T_2$ in necrotic and viable tumour tissue have given contradictory results. Jordy *et al.* (1992) studied untreated M2R mouse melanomas and found that the necrotic regions showed shorter $T_1$ than the regions of viable tissue, in agreement with the results presented here. Similarly, $T_1$ was found to decrease significantly in human cervix carcinoma during and after radiation therapy, provided that the tumours showed complete response to treatment (Santoni *et al.*, 1991). In contrast, both $T_1$ and fraction of necrotic tissue were found to increase in untreated tumours of the 13762A rat mammary carcinoma during growth (Osbakken *et al.*, 1986). Moreover, Dodd *et al.* (1989), studying the T50/80 mouse mammary carcinoma, showed that necrotic regions induced by photodynamic therapy were characterised by longer $T_1$ and $T_2$ than untreated viable tumour tissue.

The apparent discrepancy between the results from the different MRI studies may be because different types of necrosis were studied. Therapy-induced massive necrosis, particularly necrosis induced by treatment modalities causing vascular damage, e.g. photodynamic therapy and hyperthermia, is usually associated with increased oedema (Steen, 1992). Spontaneous development of massive necrosis in

**Figure 5** Synthetic spin–echo image (TR = 3300 ms and TE = 170 ms) (**a**), histology (**b**), pure $T_1$ image (**c**) and pure $T_2$ image (**d**) of the same section of a BEX-t human melanoma xenograft with a broad band of necrotic tissue centrally. The colour scales for the $T_1$ and $T_2$ images are shown in **e**.

untreated tumours can also involve development of elevated oedema, particularly in rapidly growing rodent tumours with an immature and hence fragile capillary network (Osbakken *et al.*, 1986; Belfi *et al.*, 1991). The oedema can be seen during and shortly after the development of necrosis, but it decreases gradually with time (Dodd *et al.*, 1989; Belfi *et al.*, 1991). Oedema results in an increased fraction of free to bound water and hence in lengthened $T_1$ and $T_2$ (Braunschweiger *et al.*, 1986; Steen 1992). It is possible that the effect of increased water content may overshadow the effect of increased concentrations of paramagnetic ions in some tumours with newly formed massive necrosis, i.e. tumours in which the oedema has reached its maximum level but not yet the concentration of freely dissolved paramagnetic ions. Newly formed massive necrosis, whether therapy induced or spontaneous, should therefore be distinguished from necrosis which has developed gradually during tumour growth. The MRI study of human tumour xenografts reported here refers to the latter type of necrosis. The results are thus not necessarily valid for tumours with large newly formed necrotic regions.

The present study showed that naturally occurring necrotic regions in human tumour xenografts can be detected by spin-echo MRI. If human tumour xenografts reflect tumours in man with respect to MRI properties, this observation may have significant implications for clinical oncology. Tumours with high fractions of necrotic tissue are expected to contain significant proportions of radiobiologically hypoxic cells since the viable cells adjacent to the necrotic regions in all likelihood are hypoxic (Thomlinson and Gray, 1955). This has been verified for some experimental and human tumours by using autoradiography to detect the presence of sensitiser adducts formed after administration of [$^3$H]misonidazole (Chapman, 1991). Tumours showing high fractions of necrotic tissue are thus probably resistant to radiation therapy and some forms of chemotherapy and sensitive to hyperthermia and treatment with hypoxic cell cytotoxins (Sutherland *et al.*, 1988). Consequently, the possibility exists that MRI might be used for prediction of tumour treatment response and hence for selection of the most favourable treatment protocol. Clinical studies investigating this possibility in detail are highly warranted. Moreover, tumours should be subjected to comparative MRI and histology studies following treatment with radiation, hyperthermia and cytotoxins to reveal whether therapy-induced necrosis and viable tumour tissue differ in $T_1$ and $T_2$. Detailed studies of the kinetics of therapy-induced changes in $T_1$ and $T_2$ in surviving as well as in necrotising tissue should be performed for each treatment modality. If therapy-induced necrosis can be detected by spin-echo MRI, the possibility exists that MRI also might be utilised for monitoring and early evaluation of tumour treatment response.

**Acknowledgements**
The skilful technical assistance of Berit Mathiesen and Heidi Kongshaug is gratefully acknowledged. Financial support was received from The Norwegian Cancer Society.

**References**

BAKKER CJG AND VRIEND J. (1983). Proton spin-lattice relaxation studies of tissue response to radiotherapy in mice. *Phys. Med. Biol.*, **28**, 331-340.

BARNARD AM, DE CERTAINES JD, DELAVAL PH, LOUVET M AND COETMOUR D. (1986). Histological explanation of proton $T_1$ and $T_2$ variations in lung tumors. In *Magnetic Resonance in Cancer*, Allen PS, Boisvert DPJ and Lentle BC (eds) pp. 49-51. Pergamon Press: Toronto.

BELFI CA, MEDENDORP SV AND NGO FQH. (1991). The response of the KHT sarcoma to radiotherapy as measured by water proton NMR relaxation times: relationships with tumor volume and water content. *Int. J. Radiat. Oncol. Biol. Phys.*, **20**, 497-507.

BLOEMBERGEN N, PURCELL EM AND POUND RV. (1948). Relaxation effects in nuclear magnetic resonance absorption. *Phys. Rev.*, **73**, 679-712.

BRAUNSCHWEIGER PE, SCHIFFER LM AND FURMANSKI P. (1986). $^1$H NMR relaxation times and water compartmentalization in experimental tumor models. *Magn. Reson. Imaging*, **4**, 335-342.

CHAPMAN JD. (1991). Measurement of tumor hypoxia by invasive and non-invasive procedures: a review of recent clinical studies. *Radiother. Oncol.*, **20** (Suppl.), 13-19.

DEJORDY JO, BENDEL P, HOROWITZ A, SALOMON Y AND DEGANI H. (1992). Correlation of MR imaging and histologic findings in mouse melanoma. *J. Magn. Reson. Imaging*, **2**, 695-700.

DENEKAMP J. (1993). Review article: angiogenesis, neovascular proliferation and vascular pathophysiology as targets for cancer therapy. *Br. J. Radiol.*, **66**, 181-196.

DODD NJF, MOORE JV, POPPITT DG AND WOOD B. (1989). *In vivo* magnetic resonance imaging of the effects of photodynamic therapy. *Br. J. Cancer*, **60**, 164-167.

ENGLUND E, BRUN A, GYÖRFFY-WAGNER Z, LARSSON EM AND PERSSON B. (1986). Relaxation times in relation to grade of malignancy and tissue necrosis in astrocytic gliomas. *Magn. Reson. Imaging*, **4**, 425-429.

HAWKINS RA AND PHELPS ME. (1988). PET in clinical oncology. *Cancer Metastasis Rev.*, **7**, 119-142.

JAIN RK. (1988). Determinants of tumor blood flow: a review. *Cancer Res.*, **48**, 2641-2658.

NEGENDANK W, CORBETT T, CROWLEY M AND KELLOGG C. (1991). Evidence for a contribution of paramagnetic ions to water proton spin-lattice relaxation in normal and malignant mouse tissues. *Magn. Reson. Med.*, **18**, 280-293.

OLSEN DR, LYNG H, SOUTHON TE AND ROFSTAD EK. (1994). $^{31}$P-nuclear magnetic resonance spectroscopy *in vivo* of four human melanoma xenograft lines: spin-lattice relaxation times. *Radiother. Oncol.*, **32**, 54-62.

OSBAKKEN MD, KREIDER JW AND TACZANOWSKY P. (1986). Nuclear magnetic resonance imaging characterization of rat mammary tumor. *Magn. Reson. Med.*, **3**, 1-9.

ROFSTAD EK, WAHL A, STOKKE T AND NESLAND JM. (1990). Establishment and characterization of six human melanoma xenograft lines. *APMIS*, **98**, 945-953.

ROFSTAD EK, STEINSLAND E, KAALHUS O, CHANG YB, HØVIK B AND LYNG H. (1994). Magnetic resonance imaging of human melanoma xenografts *in vivo*: proton spin-lattice and spin-spin relaxation times versus fractional tumour water content and fraction of necrotic tumour tissue. *Int. J. Radiat. Biol.*, **65**, 387-402.

SANTONI R, BUCCIOLINI M, CHIOSTRINI C, CIONINI L AND RENZI R. (1991). Quantitative magnetic resonance imaging in cervical carcinoma: a report on 30 cases. *Br. J. Radiol.*, **64**, 498-504.

SILLERUD LO, FREYER JP, NEEMAN N AND MATTINGLY MA. (1990). Proton NMR microscopy of multicellular tumor spheroid morphology. *Magn. Reson. Med.*, **16**, 380-389.

STEEN RG. (1992). Edema and tumor perfusion: characterization by quantitative $^1$H MR imaging. *Am. J. Roentgenol.*, **158**, 259-264.

SUTHERLAND RM, RASEY JS AND HILL RP. (1988). Tumor biology. *Am. J. Clin. Oncol.*, **11**, 253-274.

THOMLINSON RH AND GRAY LH. (1955). The histological structure of some human lung cancers and the possible implications for radiotherapy. *Br. J. Cancer*, **9**, 539-549.

VAN BRUGGEN N, SYHA J, BUSZA AL, KING MD, STAMP GWH, WILLIAMS SR AND GADIAN DG. (1990). Identification of tumor hemorrhage in an animal model using spin echoes and gradient echoes. *Magn. Reson. Med.*, **15**, 121-127.

VAUPEL P, KALLINOWSKI F AND OKUNIEFF P. (1989). Blood flow, oxygen and nutrient supply, and metabolic microenvironment of human tumors. *Cancer Res.*, **49**, 6449-6465.

WOODRUFF WW, DJANG WT, McLENDON RE, HEINZ ER AND VOORHEES DR. (1987). Intracerebral malignant melanoma: high-field MR imaging. *Radiology*, **165**, 209-213.